# Intrahepatic Cholestasis in a Pregnant Patient With Sickle Cell Disease: A Case Report

**DOI:** 10.7759/cureus.60611

**Published:** 2024-05-19

**Authors:** Yamini Sundara Priya Dasiah, Martina Saeid, Fatmaelzahraa Ahmed

**Affiliations:** 1 Internal Medicine, New Medical Center (NMC) Royal Women's Hospital, Abu Dhabi, ARE; 2 Obstetrics and Gynaecology, New Medical Center (NMC) Royal Women's Hospital, Abu Dhabi, ARE

**Keywords:** intrahepatic cholestasis of pregnancy in scd, hepatic complication in scd, acute sickle hepatic crisis, obstetric cholestasis, sickle cell intrahepatic cholestasis

## Abstract

Sickle cell intrahepatic cholestasis (SCIC) is a potentially fatal complication of sickle cell disease (SCD) with a high mortality rate, observed mainly in patients with homozygous SCD. Intrahepatic cholestasis of pregnancy is a known complication in pregnancy and usually presents in the late second or third trimester with itching, elevated bile acids, and elevated liver enzymes. Intrahepatic cholestasis in a pregnant patient with homozygous SCD is a rare occurrence. We present the case of a patient who was diagnosed with homozygous SCD during her second pregnancy and developed cholestasis with abnormal levels of liver enzymes at 25 weeks gestation, requiring delivery at 30 weeks gestation due to very high bile acid and liver enzyme levels. The patient was successfully managed.

## Introduction

Sickle cell intrahepatic cholestasis (SCIC) is a severe variant of sickle cell hepatic crisis. It is an uncommon but severe complication seen in patients with homozygous sickle cell disease (SCD) and sickle hemoglobin (HbS)/beta thalassemia. It is likely due to disseminated sickling within hepatic sinusoids, which leads to vaso-occlusion, hepatic ischemia, and possible evolution into multiorgan failure [1-3]. Hypoxic damage leads to the ballooning of hepatocytes and intracanalicular cholestasis. This complication, usually observed in patients with homozygous SCD, has a high mortality rate. Clinically, it can have a wide range of presentations, from mild jaundice to fulminant hepatic failure. It typically manifests as right upper quadrant pain, hepatomegaly, elevated bilirubin and liver enzymes, and a deranged coagulation profile. It can progress to multiorgan failure with encephalopathy and renal failure [4]. Initial management can be supportive with simple blood transfusions but may require exchange transfusions in severe cases [5].

Intrahepatic cholestasis of pregnancy (ICP) is characterized by pruritus accompanied by elevated bile acids and abnormal liver enzymes and usually occurs in the late second or third trimester of pregnancy, rapidly resolving after delivery. The incidence varies based on geographic location and ethnicity, with the highest incidence in South America and lower rates in Europe (about 1%) [6]. It has a complex etiology, including interactions between genetic, endocrine, and environmental factors [7].

Although maternal outcomes are generally favorable, fetal morbidity and mortality rise with ICP, including rates of preterm birth and intrauterine fetal death. Sickle cell disease in pregnancy can be associated with both fetal and maternal complications, including intrauterine growth restriction, fetal death, preterm labor, preeclampsia, and thromboembolic events. There is also an increased risk of infections, particularly urinary tract infections, pneumonia, and puerperal sepsis. Crises complicate around 35% of pregnancies in women with SCD. Here, we present a rare case of a pregnant patient with homozygous SCD with intrahepatic cholestasis who was managed with blood transfusions and had a safe outcome.

## Case presentation

A 26-year-old female patient from Nigeria, G2P1, presented to the internal medicine department at 15 weeks gestation with severe anemia. Her first pregnancy occurred seven years earlier and was uneventful. In this pregnancy, her initial hemoglobin level at 15 weeks gestation was 6.4 g/dL with ferritin of 200 ng/mL, and hemoglobin electrophoresis showed findings suggestive of SCD with HbS at 81.9% (Table 1). The sickling test was positive, and a peripheral smear indicated SCD. Reticulocyte count was elevated, with mildly elevated indirect bilirubinaemia. There was no history suggestive of painful crises in the past. The patient was initially managed with three packed red blood cell (RBC) transfusions.

**Table 1:** Hemoglobin fraction

Hemoglobin fraction	Result	Reference range
Hemoglobin A	0.0	96.7% to 97.8 %
Hemoglobin F	15.2	≤0.5 %
Hemoglobin A2	2.9	2.2% to 3.2 %
Hemoglobin S	81.9	0.0% to 0.0 %

At 24 weeks gestation, the patient presented with generalized pruritus and jaundice. Bilirubin was elevated with elevated liver enzymes. The laboratory data at this time were as follows: total bilirubin 31 µmol/L with direct bilirubin at 9 µmol/L; bile acid 22.4 µmol/L; aspartate aminotransferase (AST) 280 U/L; alanine aminotransferase (ALT) 214 U/L with normal prothrombin time and partial thromboplastin time; and serum lactate dehydrogenase was 489 U/L. Abdominal ultrasound showed mild hepatomegaly with no evidence of extrahepatic cholestasis, but gallstones were present. Other causes of jaundice and liver enzyme elevation were ruled out. Tests for antinuclear antibody, anti-mitochondrial antibody, and anti-hepatitis E antibody were negative. Consultations were sought from hematology and gastroenterology specialists.

Bilirubin levels progressively increased to 35 µmol/L with direct bilirubin at 10 µmol/L, AST at 218 U/L, ALT at 269 U/L, alkaline phosphatase (ALP) at 109 U/L at 28 weeks gestation. One unit of packed RBC was given at 28 weeks gestation, as the patient’s hemoglobin was 8.3 g/dL. Although her parameters worsened, the patient did not experience any symptoms of nausea or vomiting but had mild, nonspecific abdominal pain. Treatment with ursodeoxycholic acid was initiated at 25 weeks gestation, given the possibility of obstetric cholestasis. The possibility of SCIC was also considered, and the patient was closely monitored.

At 30 weeks gestation, total bilirubin increased to 79 µmol/L and direct bilirubin was 40 µmol/L with AST at 639 U/L, ALT at 676 U/L, and ALP at 143 U/L. Bile acid increased to 105 µmol/L, and hemoglobin was 8.6 g/dL. The patient was clinically stable and did not exhibit any nausea, abdominal pain, or bleeding manifestations, and there was no change in sensorium. Renal parameters were also normal. The evolution of liver parameters and bile acid is shown in Table 2.

**Table 2:** Progressive change in lab parameters AST: Aspartate aminotransferase, ALT: Alanine aminotransferase, ALP: Alkaline phosphatase

Parameters	15 weeks	24 weeks	26 weeks	28 weeks	30 weeks	Postnatal	1-week postnatal	Reference range
Total bilirubin	36.2	31.9	43.7	35.1	79.4	73	37.8	2–21 µmol/L
Direct bilirubin	9.1	9.1	23	10.3	40.7	31	11.1	0.3–4 µmol/L
Indirect bilirubin		22.8	20.7	24.7	38.7	41	26.7	0.15–4 µmol/L
AST		280	432	218	639	255	56	<35 U/L
ALT		214	310	269	676	351	68	<35 U/L
ALP		110	132	109	143	122	112	30–120 U/L
Bile acid		22.4		15.3	105		16.5	2–10 µmol/L

An emergency cesarean section was performed at 30+ weeks gestation because of very high bile acid levels and a significant elevation in liver enzymes. Two units of packed RBCs were administered during delivery. Supportive care was provided, and the patient made an uneventful recovery. Liver enzymes and bile acid levels improved over a period of one to two weeks. Renal parameters remained normal throughout the pregnancy.

## Discussion

In this paper, we present the case of a pregnant patient who was newly diagnosed with SCD during her second pregnancy and subsequently developed features of intrahepatic cholestasis, a potentially fatal complication of SCD. Although the exact etiology of SCIC is not fully understood, it is believed to be secondary to sickled RBCs adhering to the vascular endothelium of hepatic sinusoids, leading to ischemia and ballooning of hepatocytes, resulting in biliary stasis. Acute intrahepatic cholestasis in SCD differs from other forms of sickle cell hepatopathy in that it tends to be more severe and is often accompanied by encephalopathy, coagulopathy, and renal failure [8].

Patients typically present with right upper abdominal pain, fever, leukocytosis, and hyperbilirubinemia, with markedly elevated liver enzymes reflecting ischemic injury. Coagulopathy and renal failure may develop, likely secondary to sickle cell nephropathy or in response to multiorgan failure with hypotension [9]. Ahn et al. reported on cases of SCIC, categorizing patients into two groups based on disease severity. Group 1 had less severe disease, with a mortality rate of 4%, while group 2 had more severe disease, with a mortality rate of 64% [10].

In our case, the diagnosis of SCIC is suspected due to the patient's diagnosis of homozygous SCD with a high HbS level, coupled with the sudden onset of generalized itching, hyperbilirubinemia, and abnormal liver enzyme levels. Liver enzymes were significantly elevated, with AST reaching 639 U/L and ALT 676 U/L, along with a total bilirubin of 83 µmol/L and direct bilirubin of 46.6 µmol/L. Bile acid levels also rose progressively, going from 22 µmol/L at 24 weeks gestation to 105 µmol/L at 29 weeks gestation.

Despite the presence of gallstones, no evidence of cholecystitis or choledocholithiasis was found. Other conditions, such as acute viral hepatitis, autoimmune hepatitis, and hemochromatosis, were ruled out. The main differential diagnosis was obstetric cholestasis, or ICP, given the patient's symptoms of pruritus and progressive elevation of bile acid levels. The significance of elevated bile acids in SCIC is not well defined, and acute fatty liver disease during pregnancy was also considered.

The hematologist considered exchange transfusions due to the possibility of SCIC, but as the patient did not exhibit features of liver failure or renal dysfunction and clotting parameters remained normal, conservative management with blood transfusions was pursued. The patient received three units of packed RBCs at 17 weeks gestation, resulting in an improvement in hemoglobin levels to 9.8 g/dL. However, her hemoglobin subsequently decreased to 8.3 g/dL, necessitating the administration of another two units during delivery. Due to the progressive elevation of liver enzymes and bile acids, the patient underwent delivery at 30 weeks gestation.

## Conclusions

Sickle cell intrahepatic cholestasis represents a rare yet life-threatening complication of SCD, warranting consideration in any patient with homozygous SCD who presents with a sudden onset of elevated liver enzymes accompanied by abdominal pain and high bilirubin levels. The patient described was diagnosed with homozygous SCD during her second pregnancy and began experiencing symptoms of itching and mild, nonspecific abdominal pain starting at 24 weeks gestation, along with elevated liver enzymes and bile acids. We deliberated between the possibilities of obstetric cholestasis and SCIC. Given the patient's stable clinical condition, we initially managed her condition with blood transfusions and ursodeoxycholic acid, closely monitoring her progress. However, as her liver parameters deteriorated, necessitating intervention, the patient underwent delivery at 30 weeks gestation. Fortunately, the patient experienced an uneventful recovery post-delivery. This case is presented because of its rarity and the challenges associated with its diagnosis, which may not be readily apparent.
